# Predicting HIV Pre-exposure Prophylaxis Efficacy for Women using a Preclinical Pharmacokinetic-Pharmacodynamic *In Vivo* Model

**DOI:** 10.1038/srep41098

**Published:** 2017-02-01

**Authors:** Angela Wahl, Phong T. Ho, Paul W. Denton, Katy L. Garrett, Michael G. Hudgens, Glenn Swartz, Cynthia O’Neill, Fulvia Veronese, Angela D. Kashuba, J. Victor Garcia

**Affiliations:** 1 Division of Infectious Diseases, Center for AIDS Research, University of North Carolina at Chapel Hill, School of Medicine, Chapel Hill, 27599, United States of America; 2 Division of Pharmacotherapy and Experimental Therapeutics, Eshelman School of Pharmacy, University of North Carolina at Chapel Hill, Chapel Hill, 27599, United States of America; 3 Department of Biostatistics, Gillings School of Global Public Health, University of North Carolina at Chapel Hill, Chapel Hill, 27599, United States of America; 4 Advanced Bioscience Laboratories, Rockville, 20850, United States of America; 5 Prevention Sciences Program, Division of AIDS, National Institute of Allergy and Infectious Diseases, National Institutes of Health, Bethesda, 20852, United States of America

## Abstract

The efficacy of HIV pre-exposure prophylaxis (PrEP) relies on adherence and may also depend on the route of HIV acquisition. Clinical studies of systemic tenofovir disoproxil fumarate (TDF) PrEP revealed reduced efficacy in women compared to men with similar degrees of adherence. To select the most effective PrEP strategies, preclinical studies are critically needed to establish correlations between drug concentrations (pharmacokinetics [PK]) and protective efficacy (pharmacodynamics [PD]). We utilized an *in vivo* preclinical model to perform a PK-PD analysis of systemic TDF PrEP for vaginal HIV acquisition. TDF PrEP prevented vaginal HIV acquisition in a dose-dependent manner. PK-PD modeling of tenofovir (TFV) in plasma, female reproductive tract tissue, cervicovaginal lavage fluid and its intracellular metabolite (TFV diphosphate) revealed that TDF PrEP efficacy was best described by plasma TFV levels. When administered at 50 mg/kg, TDF achieved plasma TFV concentrations (370 ng/ml) that closely mimicked those observed in humans and demonstrated the same risk reduction (70%) previously attained in women with high adherence. This PK-PD model mimics the human condition and can be applied to other PrEP approaches and routes of HIV acquisition, accelerating clinical implementation of the most efficacious PrEP strategies.

In 2012, Truvada^®^, a fixed-dose combination tablet consisting of two nucleoside reverse transcriptase inhibitors (NRTIs), tenofovir disoproxil fumarate (TDF) and emtricitabine (FTC), received FDA approval to be used as pre-exposure prophylaxis (PrEP) for HIV prevention in high-risk individuals. Truvada^®^ received this licensing on the basis of a 44–75% decrease in HIV incidence in clinical trials of serodiscordant heterosexual couples (Partners PrEP) and men who have sex with men (MSM) (iPrEX)^1^,^2^.

Two large Phase III HIV prevention trials conducted in women, FEM-PrEP and VOICE, failed to show HIV prevention efficacy for TDF taken with or without FTC^3^,^4^, whereas women in a third clinical trial, Partners PrEP, had a high degree of protection^2^. These results were attributed to differing rates of adherence in the studies. However, the degree of adherence needed to effectively prevent HIV infection may be dependent on the route of acquisition as evidenced by recent data from the iPrEX clinical trial demonstrating that two doses of Truvada^®^ per week were sufficient to reduce rectal HIV acquisition in MSM^5^. This same level of adherence failed to protect women against vaginal HIV acquisition in FEM-PrEP and VOICE^3^,^4^.

Together, these clinical trial data indicate the importance of understanding the relationship between drug concentrations at sites of HIV acquisition (pharmacokinetics [PK]) and efficacy (pharmacodymamics [PD]) in men and women for different PrEP regimens. Understanding antiretroviral (ARV) concentrations in various fluids and tissues relevant to HIV acquisition after clinical dosing has occasionally been included in early prevention product trials, but typically has progressed in parallel with larger efficacy studies. Mostly, it is addressed only as a follow-up analysis of samples collected from these studies, and in many instances, only plasma drug concentrations were measured. Although this limited PK analysis can be useful in understanding post trial outcomes, the field needs improved pre-Phase III methods to better inform effective trial design and more accurately predict outcomes.

This need was identified by the HIV Prevention Pharmacology Best Practices Working Group (BPWG), supported by the Division of AIDS (DAIDS) at the National Institute of Allergy and Infectious Disease (NIAID) and the Bill and Melinda Gates Foundation (BMGF). In particular, dose ranging evaluations in animal models were identified as critical to determining whether predictive or relational PK-PD models could be established to inform dose selection in human studies^6^.

In bone marrow/liver/thymus (BLT) humanized mice, the female reproductive tract (FRT) and gastrointestinal tract are reconstituted with human immune cells which can then be infected with HIV^7^,^8^,^9^,^10^,^11^,^12^,^13^,^14^,^15^,^16^,^17^,^18^. BLT mice have been extensively utilized as an *in vivo* preclinical animal model to explore the efficacy of systemic and topical ARV strategies to prevent vaginal HIV acquisition^7^,^8^,^9^,^10^,^11^,^14^. Approaches have included topical tenofovir (TFV) and systemic TDF administered in combination with FTC^8^,^9^. However, BLT mice are bioengineered individually and the *in situ* development of a human immune system in each animal occurs over a period of several weeks. Comparatively, other related “off the shelf” strains like BALB/c mice are more readily available and less costly, making them appealing to use in preclinical models. BALB/c mice, however, lack human immune cells and thus are not susceptible to HIV infection. In order to use BALB/c mice and maximize resource allocation, a discernable dose-exposure relationship between BLT and BALB/c mice is needed. Therefore, we designed an investigation to understand the PK-PD relationship of systemically administered TDF in protecting BLT mice against a vaginal HIV challenge.

We determined concentrations (PK) of TFV and its active intracellular metabolite (TFV diphosphate [TFVdp]) in BLT and BALB/c mice, and established the degree of protection (PD) in BLT mice. TDF PrEP prevented vaginal HIV acquisition in a dose-dependent manner in BLT mice. PK-PD modeling of TFV in plasma, FRT tissue, cervicovaginal lavage (CVL) fluid and TFVdp in FRT tissue revealed that TDF PrEP efficacy was best described by plasma TFV levels. When administered at 50 mg/kg, TDF achieved plasma TFV concentrations similar to those observed in humans and demonstrated the same risk reduction (~70%) attained in women with high adherence in Partners PrEP^19^.

## Results

### Dose dependence of TDF PrEP activity during vaginal HIV challenge in BLT humanized mice

**Figure 1:**
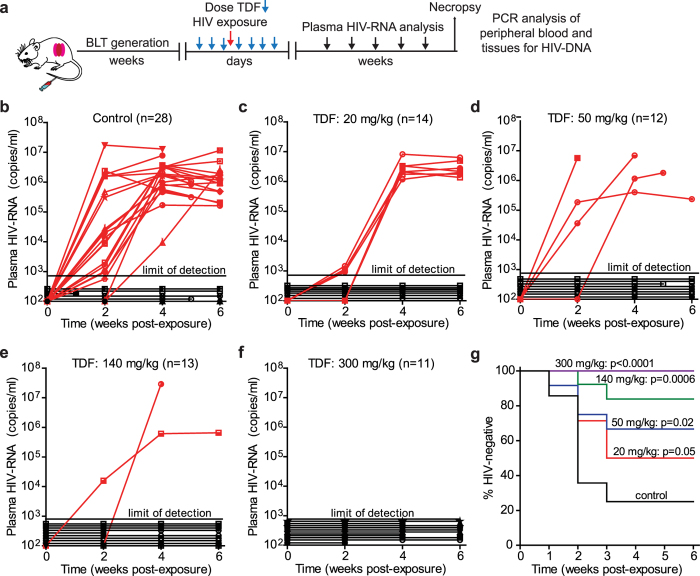
*In vivo* efficacy of systemic TDF PrEP for vaginal HIV acquisition. (**a**) The efficacy of systemic TDF PrEP was evaluated in BLT humanized mice. BLT mice were administered TDF systemically once daily for 7 consecutive days and challenged vaginally with HIV-1_JR-CSF_ 3 h after the third TDF dose. Following exposure, HIV-RNA levels were monitored longitudinally in peripheral blood plasma. BLT mice with detectable levels of plasma HIV-RNA are shown in red and mice with no detectable HIV-RNA in plasma are shown in black. In panels B-F HIV-RNA levels in plasma are shown for (**b**) control (no TDF treatment) BLT mice (n = 28) and BLT mice dosed with (**c**) 20 mg/kg (n = 14), (**d**) 50 mg/kg (n = 12), (**e**) 140 mg/kg (n = 13), and (**f**) 300 mg/kg (n = 11) TDF. The assay limit of detection (750 HIV-RNA copies/ml) is shown with a dashed line (**b**–**f**). (**g**) A Kaplan Meier plot depicts the percent of HIV-negative mice in each TDF PrEP group. A log-rank Mantel-Cox test was used to compare the percent of HIV-negative mice between groups of control mice and mice that received TDF PrEP. Mouse vector art authored by Gwilz (https://commons.wikimedia.org/wiki/File%3AVector_diagram_of_laboratory_mouse_(black_and_white).svg).

TDF (20, 50, 140, or 300 mg/kg) was administered to BLT mice systemically once daily for seven consecutive days. BLT mice were exposed vaginally to HIV 3 h after the third TDF dose (Fig. 1a) and then received four additional daily doses of TDF to reproduce current clinical trials of systemic HIV PrEP in which study participants receive daily ARVs until testing positive for HIV. As a positive control for vaginal HIV acquisition, untreated (no TDF administered) BLT mice were exposed once vaginally to HIV. Following HIV exposure, peripheral blood plasma HIV-RNA levels in BLT mice were monitored longitudinally with a real-time PCR viral load assay. At necropsy, the presence of HIV-DNA in peripheral blood and tissues was determined with real-time PCR. Protection was defined as the absence of detectable HIV-RNA in plasma at all time points analyzed and the absence of detectable HIV-DNA in peripheral blood and tissues at necropsy.

In the absence of TDF treatment, HIV-RNA was readily detected in the plasma of 75% (21/28) of control animals exposed vaginally to HIV (Fig. 1b and Table 1). Plasma HIV-RNA was detected in the vast majority of HIV-infected BLT mice by two weeks post-exposure (19/21) and in all HIV-infected mice by 4 weeks post-exposure (Fig. 1b). HIV-RNA was detected in the plasma of only 50% (7/14), 33% (4/12), and 15% (2/13) of BLT mice administered 20, 50, and 140 mg/kg TDF, respectively (Fig. 1c–f and Table S1). No HIV-RNA was detected in the plasma of any animal that received 300 mg/kg TDF at all time points analyzed up to 6 weeks post-exposure (Fig. 1f and Table S1). HIV transmission was significantly reduced in BLT mice administered 50 mg/kg (p = 0.02), 140 mg/kg (p = 0.0006), or 300 mg/kg (p < 0.0001) TDF (Fig. 1c–g and Table 1). Thus the risk reduction in HIV acquisition increased as the systemic dose of TDF increased (Table 1). The majority (8/13) of BLT mice which acquired HIV despite TDF administration had detectable HIV-RNA in their plasma by two weeks post-exposure.

The absence of HIV infection in BLT mice without detectable HIV-RNA in plasma was confirmed at necropsy by determining the presence of HIV-DNA in peripheral blood and tissues of BLT mice with real-time PCR (Table S1). No HIV-DNA was detected in peripheral blood or tissues analyzed from BLT mice with undetectable levels of HIV-RNA in plasma confirming protection from infection. In contrast, HIV-DNA was readily detected in peripheral blood and tissues analyzed from HIV-infected BLT mice that received TDF and had detectable HIV-RNA.

### Comparative TFV and TFVdp concentrations in BLT and BALB/c mice

**Figure 2:**
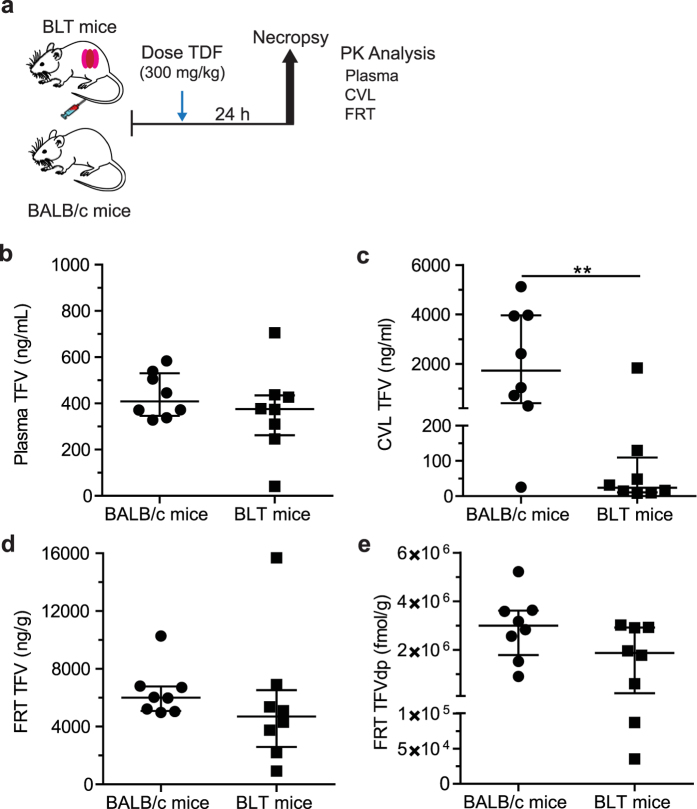
Pharmacokinetics of TFV in BALB/c and BLT mice. (**a**) BALB/c (n = 8) and BLT (n = 8) mice were administered a single dose of 300 mg/kg TDF and the concentrations of TFV (plasma, CVL, and the FRT) and TFVdp (FRT) measured 24 h later. The concentration of TFV present in (**b**) plasma, (**c**) CVL, and (**d**) the FRT of BALB/c and BLT mice. (**e**) The concentration of TFVdp in the FRT of BALB/c and BLT mice. (**b**–**e**) Shown are the median TFV and TFVdp concentrations (horizontal line) and interquartile range (vertical lines). An exact Mann-Whitney test was used to compare the concentrations of TFV and TFVdp between BALB/c and BLT mice (*p < 0.05, **p < 0.01). Mouse vector art authored by Gwilz (https://commons.wikimedia.org/wiki/File%3AVector_diagram_of_laboratory_mouse_(black_and_white).svg).

BALB/c mice are more readily available and less costly than BLT mice, and the immune deficient mouse strains utilized to generate them, but they lack human immune cells and thus are not susceptible to HIV infection. To determine if BALB/c mice could be established as a surrogate *in vivo* model for PK analysis, TFV concentrations in plasma, CVL, and FRT tissue and TFVdp concentrations in FRT tissue 24 h following a single 300 mg/kg dose of TDF to BALB/c (n = 8) and BLT (n = 8) mice were determined (Fig. 2a and Tables S2 and S3). No significant difference was observed in (median [min-max]) plasma TFV concentrations between BALB/c (409 [328–584] ng/ml) and BLT (376 [41.5–705] ng/ml) mice (p = 0.43) (Fig. 2b and Table S2), with median concentrations differing by <9%. CVL TFV concentrations were significantly higher (p = 0.007) in BALB/c mice (1,725 [25.2–5120] ng/ml) compared to that of BLT mice (23.8 [8.7–1830] ng/ml) (Fig. 2c and Table S2). Median concentrations differed by ~200%. Although there was no significant difference in the concentration of TFV in the FRT tissue of BALB/c (6,005 [4,982–10,267] ng/g) and BLT (4,705 [923–15,680] ng/g) mice (p = 0.23) (Fig. 2d and Table S2) or in the concentration of TFVdp in BALB/c (3,000,089 [907,308–5,219,985] fmol/g) and BLT (1,870,182 [35,336–3,023,969] fmol/g) mice (p = 0.10) (Fig. 2e and Table S2), the median concentrations for TFV and TFVdp in the FRT tissue differed by 24% and 46% in BLT mice compared to BALB/c mice, respectively. Therefore, the conversion factor calculated (by dividing median concentrations) to modify the concentrations in BALB/c mice to BLT mouse exposure in the FRT tissue was 0.78 for TFV and 0.62 for TFVdp. A CVL conversion factor was not generated due to large (and expected) variability in sample concentrations.

### PK assessment of tenofovir in BALB/c mice

**Figure 3:**
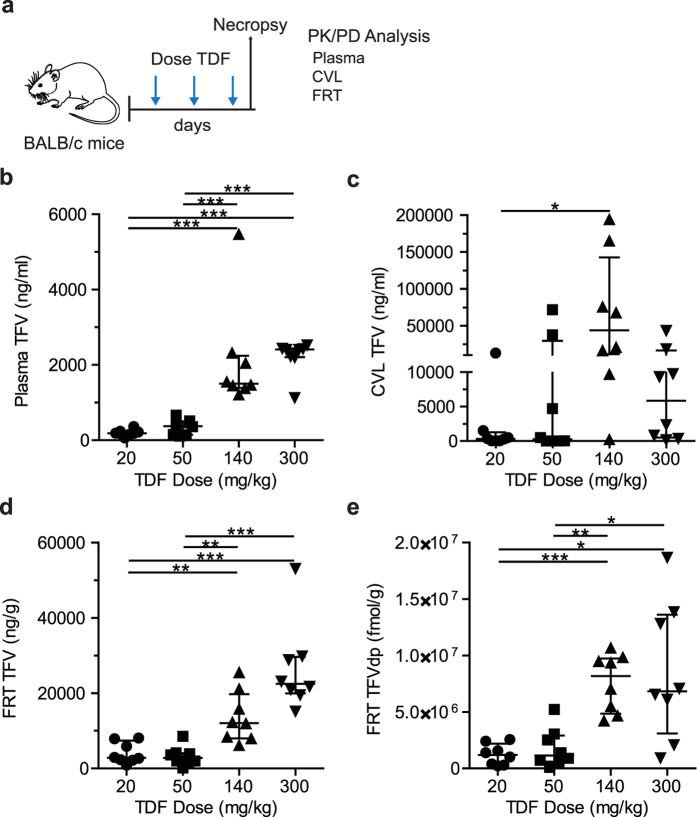
Pharmacokinetics of TFV in peripheral blood plasma, CVL and the FRT. (**a**) BALB/c mice were administered TDF once daily for three days and the concentrations of TFV (plasma, CVL, and FRT) and TFV-DP (FRT) measured 3 h after the third TDF dose in order to determine the concentrations of TFV and TFVdp present systemically and locally at the time of vaginal HIV challenge in our study. TFV concentration in (**b**) plasma, (**c**) CVL, and (**d**) the FRT of BALB/c mice dosed with 20 mg/kg (n = 8), 50 mg/kg (n = 8), 140 mg/kg (n = 8), and 300 mg/kg (n = 8) TDF. (**e**) TFVdp concentration in the FRT of BALB/c mice dosed with 20 mg/kg (n = 8), 50 mg/kg (n = 8), 140 mg/kg (n = 8), and 300 mg/kg (n = 8) TDF. (**b**–**e**) Shown are the median TFV and TFVdp concentrations (horizontal line) and interquartile range (vertical lines). Dashed lines represent the 95% confidence interval. An exact Wilcoxon rank sum test with Bonferroni correction for multiple comparisons was used to compare the concentrations of TFV and TFVdp between mice dosed with 20 mg/kg, 50 mg/kg, 140 mg/kg, and 300 mg/kg TDF (*p < 0.05, **p < 0.01, ***p < 0.001, ****p < 0.0001). Mouse vector art authored by Gwilz (https://commons.wikimedia.org/wiki/File%3AVector_digram_of_laboratory_mouse_(black_and_white).svg).

**Figure 4:**
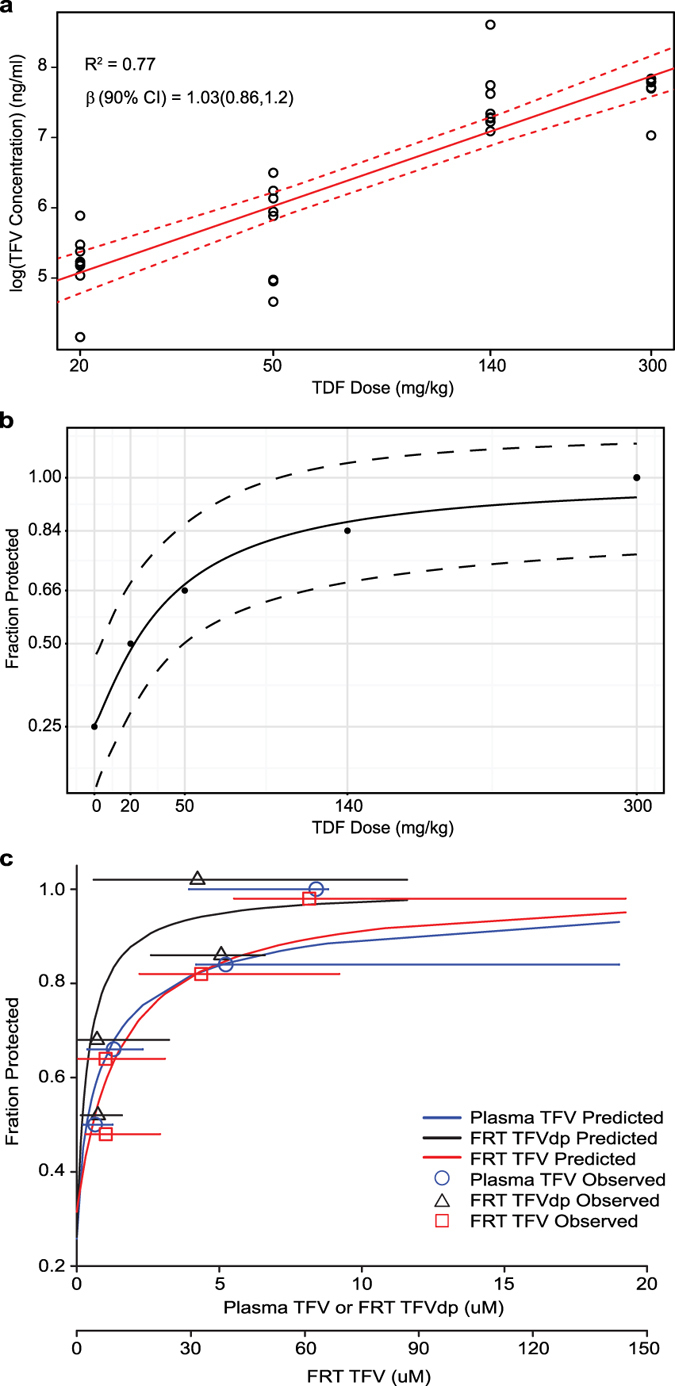
PK-PD modeling of systemic TDF PrEP. (**a**) Log tenofovir (TFV) plasma concentration to dose. Individual concentrations are displayed as open circles and the regression line is in red. (**b**) PD effect by dose. Curve estimating fraction of protection conferred by increasing dose. Observed data points are overlaid as closed circles. Fraction protected is on the y-axis with dose (mg/kg) on the x-axis. Dashed lines represent the 95% confidence interval. (**c**) PD effect by concentration curves estimating fraction of protection conferred by concentrations of TFV in plasma and the FRT as well as TFVdp in the FRT. Observed data (median [range]) are overlaid symbols with error bars. FRT TFV concentrations are scaled by 0.78 and TFVdp concentrations are scaled by 0.62. Values are offset along the y-axis for visual purposes. Fraction protected is along the y-axis and concentrations are on the x-axes.

After determining the relationship between drug concentrations in BALB/c and BLT mice, the PK of TFV in BALB/c mice systemically administered daily doses of 20, 50, 140, or 300 mg/kg TDF was evaluated (Fig. 3a). Molar conversion of TFVdp in FRT tissue was also calculated (fmol/g TFVdp ÷ fmol/g TFV). Significantly higher concentrations of TFV were detected in the plasma, CVL, and FRT tissue of mice dosed with 140–300 mg/kg TDF (Fig. 3b–d and Table S4) compared to those dosed with 20–50 mg/kg. The concentration of TFVdp in FRT tissue was also significantly higher in mice dosed with 140–300 mg/kg TDF compared to mice dosed with 20–50 mg/kg. However, the concentrations did not increase linearly with increasing dose across all matrices. Dose proportionality was declared for TFV plasma concentrations (Fig. 4a), since the slope (90% CI) of the linear regression model was 1.03 (0.86, 1.20) (Fig. 4a). However, concentrations of TFV and TFVdp in FRT tissue were not dose proportional (slope [90% CI]), TFV (0.86 [0.59, 1.12]) and TFVdp (0.86 [0.57, 1.15)]). TFV concentrations in CVL was also not dose proportional (1.33 [0.45, 2.21]). Variability in drug concentrations are reported as percent coefficient of variation (CV%). Plasma TFV, FRT TFV, and FRT TFVdp all had a CV% of approximately 100%. The mean (standard deviation) of TFVdp conversion in tissue was 13.1 (7.4)%.

### PK-PD analysis of tenofovir

The *in vivo* protective efficacy of TDF in BLT mice (Fig. 1) was correlated to the corresponding concentrations of TFV and/or TFVdp measured in plasma, FRT tissue, and CVL of BALB/c mice using the same TDF doses (Fig. 3). Final estimated parameters of the PD models, along with precision estimates (SE) are shown in Table 2. Precision was highest in the dose-response model (Fig. 4b) and lowest in the CVL concentration model (Table 2), coinciding with inter-individual variability in concentrations. The efficacy predicted for 140 and 300 mg/kg TDF doses were 86 and 93% (Fig. 4b), respectively, compared to the 84 and 100% observed, demonstrating good model fit. Estimated effect curves for plasma and tissue concentrations are shown in Fig. 4c. The median plasma TFV concentrations in the 20, 50, 140, and 300 mg/kg TDF dosing cohorts were predicted to provide 58, 67, 84, and 88% protection, respectively (Fig. 4c). Predicted efficacy based on median TFV concentrations was within 10%, again demonstrating good model fit. For TFVdp, median concentrations were highest in the 140 mg/kg cohort, resulting in no predicted increase in efficacy in animals dosed with 300 mg/kg TDF (Fig. 4c). Although the standard errors for all estimated parameters in tissue was <23%, there was considerable overlap in the concentrations providing 84–100% protection, since TFV and TFVdp concentrations in FRT tissue did not increase linearly with dose (Fig. 4c).

## Discussion

Worldwide, HIV/AIDS is the leading cause of death in young women^20^,^21^, making HIV prevention a critical component to their survival. In the absence of a vaccine or the elimination of high-risk behaviors, PrEP provides a viable alternative to prevent HIV acquisition. TDF, with or without FTC, showed promising efficacy in early, preclinical studies^22^,^23^. However, clinical trial results were mixed.

Three studies (Partners PrEP, VOICE, and FemPrEP), using the same daily HIV treatment dose of TDF, but enrolling different populations of HIV-uninfected women, yielded conflicting results^1^,^3^,^4^. Partners PrEP enrolled older women in a long-term serodiscordant relationship and demonstrated that TDF was 71% effective in protecting against HIV acquisition. VOICE and FemPrEP enrolled younger women, most of whom were without a steady partner, and demonstrated that TDF was not effective in protecting women from HIV infection. This difference in efficacy was largely attributed to adherence, since the protected women in Partners PrEP had double the rate of TFV detected in plasma compared to women enrolled in the VOICE and FemPrEP studies, and low rates of TFV detection were seen in the seroconverters in each study^1^,^3^,^4^.

The determination of PK-PD relationships is a fundamental component of drug development. Given the inconsistent outcomes in phase II PrEP studies, there is increased need to appropriately study PK-PD relationships with prevention products in order to inform development decisions. The HIV Prevention Pharmacology BPWG determined that one of the most critical needs in the HIV prevention field is to delineate the PK relationship between humans and animal models of HIV infection^6^. A number of key studies were identified that could potentially address this specific need. One of these is our investigation into the PK-PD relationship of TDF in BLT mice, a previously validated *in vivo* preclinical model of vaginal HIV acquisition^7^,^8^,^9^,^10^,^12^,^13^,^14^,^24^. We chose TDF due to its widespread clinical use and the unique pharmacological challenges the active intracellular metabolite provides to PK-PD modeling.

Our results demonstrated that the degree of protection conferred by daily systemic TDF in BLT mice was dose-dependent. Vaginal HIV acquisition was significantly decreased in mice receiving 50 and 140 mg/kg TDF and completely prevented in mice administered 300 mg/kg TDF. The PD model of dose and efficacy consistently predicted higher protective effects with increasing TDF doses, with parameter standard errors ≤10%. These results were paralleled by a dose proportional increase in TFV plasma concentrations and predictive efficacy similar to the dose-response model. Additionally, we saw similar rates of predicted protection based on TFV tissue concentrations in mice dosed with 140 and 300 mg/kg, but not those dosed with 20 or 50 mg/kg. Thus, across the entire dose range, plasma concentrations provided the best physiological predictor of efficacy.

Our PD models were unable to generate appreciable increases in protection with FRT TFVdp concentrations at doses above 140 mg/kg, which we attribute to concentration variability in this tissue. FRT TFVdp concentrations in BALB/c and BLT mice were higher than those reported in human and pigtail macaque vaginal tissue tissue 24 h after a single systemic dose of FTC/TDF. However, it is important to note that in our study, we measured TFVdp levels in the entire FRT (uterus, cervix, and vagina)^25^,^26^. TFV concentrations in CVL also increased with higher doses of TDF, but not in a dose proportional manner. These data are consistent with human CVL data which are known to be more variable (by up to 1 log) than blood plasma^25^. In women, penetration of TFV into vaginal and cervical tissue, and cervicovaginal secretions (CVS) has been demonstrated, as determined by the AUC ratio of tissue or fluid to plasma^27^. TFV levels in human vaginal and cervical tissue are lower than plasma^25^,^28^,^29^. Here, we observed higher TFV concentrations in FRT tissue of mice compared to plasma. In directly-aspirated CVS from women, TFV concentrations are 25% lower than plasma after multiple-dosing^30^. TFV CVL concentrations were more than 3-fold higher in BALB/c mice compared to plasma. Higher TFV concentrations in vaginal secretions compared to plasma has also been reported in pigtail macaques following systemic FTC/TDF administration^26^. However, TFV levels in CVL were significantly lower in BLT mice compared to BALB/c mice. As observed in humans, TFV levels were lower in CVL of BLT mice compared to plasma.

All together, we observed a clear trend toward greater protection with higher systemic TDF doses and higher TFV plasma concentrations. Plasma TFV concentrations agreed best with protective efficacy of TDF PrEP over the dosing range. TFV plasma concentrations most closely mimicking human data were observed in BALB/c mice dosed with 50 mg/kg TDF^31^,^32^. A 50 mg/kg dose of TDF demonstrated 70% risk reduction in BLT mice which was comparable to the 76% risk reduction observed in women in Partner’s PrEP with high adherence to TDF^19^.

Dose-ranging studies were identified as a critical need by the HIV Prevention BPWG in order to discern PK-PD relationships between animal models and humans^6^. Here, we showed a strong dose-response relationship and increasing protection with increasing TFV plasma concentrations across the entire dose range in the BLT mouse model of vaginal HIV acquisition. Peripheral blood plasma is a much more readily accessible biological matrix to sample and continually monitor in patients compared to tissue and CVS. Of note, we did not evaluate the efficacy of TDF PrEP for rectal HIV acquisition or colorectal TFV/TFVdp tissue concentrations, therefore, we cannot extrapolate our PK-PD data in this vaginal challenge model to rectal challenge. Colorectal tissue concentrations are likely different from genital concentrations, requiring different doses and concentrations to achieve protective effects. Although the protective efficacy of systemic TDF PrEP for vaginal HIV acquisition was best described by plasma TFV concentrations, the tissue distribution of other PrEP agents will need to be evaluated to determine if plasma or tissue drug concentrations are best associated with protective efficacy.

In summary, this preclinical PK-PD model mimics the human condition and provides a framework to accelerate clinical implementation of efficacious PrEP strategies by 1) assessing their penetration, distribution, and accumulation into relevant sites of HIV exposure, 2) evaluating their protective efficacy for different modes of HIV acquisition, 3) determining if plasma or tissue drug concentrations are best associated with predictive efficacy, and 4) identifying the best drug doses and dosing regimens to evaluate in clinical studies.

## Methods

### Experimental design

The objective of the study was to investigate the PK-PD relationship of TDF for PrEP in a preclinical *in vivo* BLT humanized mouse model of vaginal HIV acquisition. The PD assessment of TDF PrEP for prevention of vaginal HIV acquisition was evaluated by administering BLT mice a single intraperitoneal (IP) dose of 20, 50, 140, or 300 mg/kg TDF daily for seven consecutive days and exposing mice vaginally to HIV-1_JR-CSF_ 3 h after the third TDF dose as illustrated in Fig. 1a. A Kruskal-Wallis test using Dunn’s multiple comparisons test was performed to ensure that peripheral blood humanization levels (%hCD4^+^ T cells of live cells) between exposure groups were equivalent. Vaginal HIV exposures were performed as previously described by pipetting virus (3.5 × 10^5^ TCIU HIV-1_JR-CSF_) in vehicle (RPMI medium, 20 μl total volume) directly into the vaginal cavity of anesthetized BLT mice^7^,^8^,^9^,^10^,^11^,^12^,^13^,^14^,^24^. Following HIV exposure, peripheral blood plasma HIV-RNA levels in BLT mice were monitored longitudinally and at necropsy, the presence of HIV-DNA in peripheral blood and tissues was determined with real-time PCR as described below. Protection was defined as the absence of detectable HIV-RNA in peripheral blood plasma at all time points analyzed and the absence of detectable HIV-DNA in peripheral blood and tissues at necropsy. For the comparison of drug levels between BALB/c and BLT mice, animals were administered a single IP dose of 300 mg/kg TDF and samples collected 24 h later (Fig. 2a). For the PK assessment, BALB/c mice were administered a single daily IP dose of 20, 50, 140, or 300 mg/kg TDF for three consecutive days and necropsied 3 h after the third TDF dose (Fig. 3a) to mimic fluid and tissue concentrations at the time of HIV exposure in BLT mice. The presence of TFV and/or TFVdp was determined in peripheral blood, CVL, and FRT tissue collected at necropsy as described below.

### Generation of BLT humanized mice

BLT humanized mice were bioengineered as previously described^7^,^8^,^9^,^10^,^11^,^12^,^13^,^14^,^16^,^17^,^24^,^33^,^34^,^35^,^36^,^37^,^38^. Briefly, human fetal liver and thymus tissue (ABR Inc., Alameda, CA) were implanted under the kidney capsule of irradiated (200 rads) female NOD.Cg-Prkdc^scid^ ll2rg^tm1Wjl^/SzJ mice (NSG; The Jackson Laboratory, Bar Harbor, ME). Following tissue implantation, mice received autologous CD34^+^ hematopoietic stem cells via tail vein injection. Human immune cell reconstitution was monitored in the peripheral blood of BLT mice longitudinally by flow cytometry as previously described^7^,^8^,^9^,^10^,^11^,^12^,^13^,^14^,^16^,^17^,^24^,^33^,^34^,^35^,^36^,^37^,^38^. BALB/c is the parental strain for the immunodeficient mouse strain Cg-Prkdc^scid^ which was crossed with NOD mice to generate NOD.CB17-Prkdc^scid^/J mice. The NOD.CB17-Prkdc^scid^/J strain was then crossed with B6.129S4-*Il2rg*^*tm1Wjl*^/J mice to create NSG mice which were used for the preparation of BLT mice^39^. BLT mice and BALB/c mice (The Jackson Laboratory, Bar Harbor, ME) were maintained by the Division of Laboratory Animal Medicine at UNC-Chapel Hill according to protocols approved by the Institutional Use and Care Committee and in adherence to the NIH Guide for the Care and Use of Laboratory Animals.

### Virus

HIV-1_JR-CSF_, a CCR5-tropic early passage primary isolate was used for these experiments. HIV-1_JR-CSF_ has been well characterized for its ability to infect humanized mice after vaginal exposure in multiple studies^7^,^8^,^9^,^10^,^12^,^13^,^14^,^24^. In addition, it has been extensively used to investigate the *in vivo* efficacy of HIV prevention approaches in humanized mice^7^,^8^,^9^,^10^,^14^. Concentrated stocks of HIV-1_JR-CSF_ were prepared by transient transfection of 293 T cells and titered in triplicate on TZM-bl cells (NIH AIDS Research and Reference Reagent Program) as previously described to calculate the number of tissue culture infectious units per ml (TCIU)/ml of virus preparation^7^,^8^,^9^,^10^,^11^,^12^,^14^,^16^,^17^,^24^,^33^,^35^,^36^,^37^,^38^.

### Drug

TDF was obtained through the NIH AIDS Reagent Program, NIAID, NIH and also kindly provided by Jim Rooney at Gilead Sciences, Inc (Foster City, CA). TDF was solubilized in physiological saline (Hospira, Lake Forest, IL) at a final concentration of 10 mg/ml.

### Analysis of HIV infection in BLT mice

Following vaginal HIV exposure, HIV-RNA levels were monitored longitudinally in peripheral blood plasma of BLT mice with a real-time PCR viral load assay (limit of detection: 750 HIV-RNA copies/ml) as previously described^7^,^10^,^11^,^12^,^14^,^24^,^40^. The presence of HIV-DNA in tissues and peripheral blood cells collected from BLT mice at necropsy was determined by real-time PCR analysis of DNA extracted from mononuclear cells as previously described^7^,^10^,^11^,^12^,^14^,^24^,^40^. Prior to necropsy, animals were euthanized with an overdose of anesthesia followed by cervical dislocation. To control for the presence of amplifiable DNA extracted from human cells, the presence of human gamma globulin DNA was confirmed by real-time PCR for all samples. Protection from HIV infection was defined by the absence of detectable HIV-RNA in plasma at all time points analyzed and the absence of detectable HIV-DNA in peripheral blood cells and tissues at necropsy.

### Sample collection and handling

Peripheral blood was collected with EDTA coated capillary tubes and plasma was subsequently isolated by centrifugation (2,000 RPM for 5 min at RT). CVL were performed with sterile PBS (3 washes of 20 μl each, ~60 μl total volume) as previously described^11^,^12^,^40^. The entire FRT (vagina, cervix and uterus) was weighed and snap frozen with liquid nitrogen. All samples were stored at −80 °C until analysis.

### Quantification of TFV and TFVdp. 

Once TDF is administered, it is metabolized to TFV by hydrolases in the gut and plasma. Circulating TFV is subsequently metabolized intracellularly by cellular kinases to its active metabolite TFVdp. We quantified TFV and TFVdp in biological matrices using validated LC-MS/MS methods as previously described^41^. Quantification of TFV concentrations in plasma and CVL was performed by protein precipitation and LC-MS/MS analysis with an isotopically-labeled internal standard (^13^C TFV). TFV was eluted from a Waters Atlantis T3 (100 × 2.1 mm, 3 μm particle size) analytical column and an API-5000 triple quadrupole mass spectrometer (AB Sciex, Foster City, CA) was used to detect the analytes. Data were collected using AB Sciex Analyst Chromatography Software (Analyst version 1.6.1). The dynamic range of this assay was 2–2000 ng/mL for plasma and 1–2000 ng/mL for CVL using a 1/concentration^2^ weighted linear regression. For measuring concentrations in mucosal tissues, TFV and TFVdp was extracted from tissue homogenate by protein precipitation with isotopically-labeled internal standards (^13^C TFV and ^13^C TFVdp). TFV was eluted from a Waters Atlantis T3 (100 × 2.1 mm, 3 μm particle size) analytical column, and TFVdp was eluted from a Thermo Biobasic AX (50 × 2.1 mm, 5 μm particle size) analytical column. An API-5000 triple quadrupole mass spectrometer was used to detect all analytes. Data were collected using AB Sciex Analyst Chromatography Software (Analyst version 1.6.1). The dynamic range of this assay was 0.3–300 ng/mL of homogenate for each compound using a 1/concentration^2^ weighted linear regression. Concentrations were ultimately converted into ng/g (TFV) or fmol/g (TFVdp) tissue for final reporting.

### PK data analysis

A linear regression was fit (Eqn. 1) for the log-transformed concentration data of TFV and TFVdp to log-transformed dose in order to determine if concentrations increased in a dose proportional manner. Dose proportionality was declared if the 90% confidence interval for the slope β was contained in the interval 0.744–1.26^42^. Regression analyses were performed in RStudio^43^.


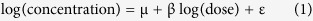


### Comparison of drug exposure between BLT and BALB/c mice

Concentrations of TFV and/or TFVdp in plasma, CVL, and FRT tissue were compared between BLT and BALB/c mice to determine the similarity in TFV and TFVdp exposure. This was done to identify whether any adjustment (or conversion) factor would need to be applied to the more frequently collected BALB/c samples to estimate the BLT concentrations when establishing the PK-PD relationship for BLT mice. Median concentrations were calculated for each matrix; if the medians between BLT and BALB/c mice differed by more than 20% an adjustment (conversion) factor to predict BLT concentrations using BALB/c concentrations was generated by dividing the respective median values. If the median values differed by less than 20%, no adjustment was made to the concentrations.

### PD model development

The concentrations of TFV and TFVdp obtained from the BALB/c mice in the PK assessment were related to the *in vivo* efficacy data from BLT mice in the PD assessment to generate predictive PK-PD models for each biological matrix. A fractional sigmoidal Hill function (Eqn. 2) was used to describe the proportion of animals protected from vaginal HIV challenge based on the concentration of TFV or TFVdp in each of the matrices. The same model was also used to describe the relationship between protection and TDF dose (dose-response model). A conversion factor (as described above) was used to adjust BALB/c concentrations to expected BLT concentrations for tissue TFV and TFVdp concentrations. In this model, E_max_ was the maximum protection and E_0_ was the baseline protection. EC_50_ was the concentration at which half-maximal efficacy occurs and H was the Hill’s coefficient. DV was the dependent variable (i.e, TFV or TFVdp concentration) being modeled. Parameters were estimated using the maximum likelihood (ML) estimation function in ADAPT5 (Biomedical Simulations Resource, Los Angeles, CA, USA). E_max_ was fixed to one for all models. H was fixed to one for TFV in tissue and CVL because the estimation routine resulted in false minima (H < 0.5). H was estimated in all other matrices. Proportional residual error variance models were used and estimated, with CV% of the assays as initial estimates.


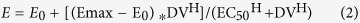


### Statistical analysis

Statistical analyses were performed in Prism, version 6 (Graph Pad, La Jolla, CA). Two-sided testing and an alpha of 0.05 was used. A log-rank Mantel-Cox test was used to compare the percent of HIV-negative BLT mice between groups of control mice and mice that received TDF PrEP. An exact Mann-Whitney U test was used to compare TFV and TVFdp concentrations between BALB/c and BLT mice. TFV and TVFdp concentrations between BALB/c mice dosed with 20, 50, 140, or 300 mg/kg TDF were compared with an exact Wilcoxon rank sum test using the Bonferroni correction for multiple comparisons.

## Additional Information

**How to cite this article:** Wahl, A. *et al*. Predicting HIV Pre-exposure Prophylaxis Efficacy for Women using a Preclinical Pharmacokinetic-Pharmacodynamic *In Vivo* Model. *Sci. Rep.* **7**, 41098; doi: 10.1038/srep41098 (2017).

**Publisher's note:** Springer Nature remains neutral with regard to jurisdictional claims in published maps and institutional affiliations.

## Supplementary Material

Supplementary Information

## Figures and Tables

**Table 1:** Protective Efficacy of TDF PrEP.

Treatment group	Total mice (n)	Infected mice (n)	Uninfected mice (n)	Risk reduction (95% CI), %	*P*
20 mg/kg	14	7	7	61% (−1.2 to 85)	0.05
50 mg/kg	12	4	8	70% (16 to 89)	0.02
140 mg/kg	13	2	11	83% (53 to 94)	0.0006
300 mg/kg	11	0	11	88% (66 to 95)	<0.0001
Control	28	21	7	NA	NA

Risk reduction: (1-Hazard Ratio) × 100. NA: not applicable.

**Table 2:** PD Model Parameters.

Parameter	Dose (mg/kg)	Plasma (ng/ml)	FRT TFV (ng/g)	FRT TFVdp (fmol/g)	CVL (ng/ml)
E_0_	0.2502 [3.67]	0.2580 [13.24]	0.3151 [7.299]	0.2622 [7.405]	0.4016 [8.918]
EC_50_	38.7 [7.628]	259 [19.6]	4101 [21.18]	6.052e5 [22.75]	238.7 [51.53]
H	1.138 [10.4]	0.7449 [14.98]	NA	NA	NA

All values are presented as mean (standard error [SE]). E^0^: baseline effect, EC_50_: concentration or dose reflecting half-maximal effect, H: Hill’s coefficient, FRT: female reproductive tract, TFV: tenofovir, TFVdp: tenofovir diphosphate, CVL: cervicovaginal lavage, and NA: not applicable.
