# Supplementary material for: Predicting HIV Pre-exposure Prophylaxis Efficacy for Women using a Preclinical Pharmacokinetic-Pharmacodynamic In Vivo Model
Source: Sci Rep. 2017 Feb 1;7:41098. doi: 10.1038/srep41098 (PMC5286499; doi:10.1038/srep41098)
Supplement: Supplementary Information [file srep41098-s1.pdf]

Predicting HIV Pre-exposure Prophylaxis Efficacy for Women using a Preclinical Pharmacokinetic-Pharmacodynamic *In Vivo* Model

Angela Wahl[1*], Phong T. Ho[1], Paul W. Denton[1+,#], Katy L. Garrett[2+], Michael G. Hudgens[3], Glenn Swartz[4], Cynthia O'Neill[4], Fulvia Veronese[5], Angela D. Kashuba[2], and J. Victor Garcia[1]

[1]Division of Infectious Diseases, Center for AIDS Research, University of North Carolina at Chapel Hill, School of Medicine, Chapel Hill, 27599, United States of America

[2]Division of Pharmacotherapy and Experimental Therapeutics, Eshelman School of Pharmacy, University of North Carolina at Chapel Hill, Chapel Hill, 27599, United States of America

[3]Department of Biostatistics, Gillings School of Global Public Health, University of North Carolina at Chapel Hill, Chapel Hill, 27599, United States of America

[4]Advanced Bioscience Laboratories, Rockville, 20850, United States of America

[5]Prevention Sciences Program, Division of AIDS, National Institute of Allergy and Infectious Diseases, National Institutes of Health, Bethesda, 20812, United States of America

[#]Current address: Aarhus University; Department of Clinical Medicine and Aarhus University Hospital-Skejby; Department of Infectious Diseases (Q), Aarhus, 8200, Denmark.

*Corresponding author: awahl@med.unc.edu

[+]These authors contributed equally to this work

Table S1. Description of BLT mice Utilized to Evaluate the Efficacy of TDF PrEP to Prevent Vaginal HIV Acquisition.

		Peripheral blood humanization	At time of necropsy							
		At time of exposure	Presence of HIV-RNA (+/-)	Presence of HIV-DNA (+/-)						
Mouse ID	TDF Dose (mg/kg)	%hCD4^{+} T Cells of Live Cells	Plasma	SPL	LN	BM	ORG	LIV	LNG	PB
TDF20-1	20	44.5	+	ND	ND	ND	ND	ND	ND	+
TDF20-2	20	45.6	+	ND	ND	ND	ND	ND	ND	+
TDF20-3	20	36.0	-	-	ND	-	-	-	-	-
TDF20-4	20	30.6	+	ND	ND	ND	ND	ND	ND	+
TDF20-5	20	41.0	-	-	ND	-	-	-	-	-
TDF20-6	20	31.2	-	-	ND	-	-	-	-	-
TDF20-7	20	27.6	-	-	ND	-	-	-	-	-
TDF20-8	20	35.6	-	-	ND	-	-	-	-	-
TDF20-9	20	25.1	-	-	ND	-	-	-	-	-
TDF20-10	20	23.3	+	ND	ND	ND	ND	ND	ND	+
TDF20-11	20	28.0	+	ND	ND	ND	ND	ND	ND	+
TDF20-12	20	25.1	-	-	ND	-	-	-	-	-
TDF20-13	20	37.1	+	ND	ND	ND	ND	ND	ND	+
TDF20-14	20	42.1	+	ND	ND	ND	ND	ND	ND	+
Mean ± SD		33.8 ± 7.5								
TDF50-1	50	34.6	-	-	-	-	-	-	-	-
TDF50-2	50	39.0	-	-	-	-	-	-	-	-
TDF50-3	50	38.3	-	-	-	-	-	-	-	-
TDF50-4	50	22.0	+	+	+	+	+	+	+	+
TDF50-5	50	21.0	-	-	-	-	-	-	-	-
TDF50-6	50	21.6	-	-	-	-	-	-	-	-
TDF50-7	50	35.1	+	+	+	+	+	+	+	+
TDF50-8	50	42.1	+	+	ND	+	+	+	+	+
TDF50-9	50	39.4	+	+	+	+	+	+	+	+
TDF50-10	50	40.8	-	-	ND	ND	-	-	ND	-
TDF50-11	50	33.3	-	-	ND	-	-	-	-	-
TDF50-12	50	42.5	-	-	ND	-	-	-	-	-
Mean ± SD		34.2 ± 8.1								
TDF140-1	140	25.4	-	-	-	-	-	-	-	-
TDF140-2	140	32.5	-	-	-	-	-	-	-	-
TDF140-3	140	26.9	-	-	-	-	-	-	-	-
TDF140-4	140	16.8	-	-	-	-	-	-	ND	-
TDF140-5	140	32.9	-	-	ND	-	-	ND	-	-
TDF140-6	140	21.3	+	ND	ND	+	+	+	+	+
TDF140-7	140	37.0	-	-	ND	-	-	-	-	-
TDF140-8	140	26.0	-	-	ND	ND	-	-	-	-
TDF140-9	140	37.5	-	-	ND	-	-	-	-	-
TDF140-10	140	44.9	-	-	ND	-	-	-	-	-
TDF140-11	140	50.7	-	-	ND	-	-	-	-	-
TDF140-12	140	30.7	+	ND	ND	ND	ND	ND	ND	+
TDF140-13	140	23.7	-	-	ND	-	-	-	-	-
Mean ± SD		31.2 ± 9.5								
TDF300-1	300	36.7	-	-	-	-	-	-	-	-
TDF300-2	300	34.2	-	-	-	-	-	-	-	-
TDF300-3	300	39.0	-	-	-	-	-	-	-	-
TDF300-4	300	60.2	-	-	-	-	-	-	-	-
TDF300-5	300	30.0	-	-	-	-	-	-	-	-
TDF300-6	300	43.4	-	-	-	-	-	-	-	-
TDF300-7	300	21.3	-	-	-	-	-	-	-	-
TDF300-8	300	35.9	-	-	-	-	-	-	-	-
TDF300-9	300	32.0	-	-	-	-	-	-	-	-
TDF300-10	300	25.5	-	-	-	-	-	-	-	-
TDF300-11	300	29.9	-	-	-	-	-	-	-	-
Mean ± SD		35.3 ± 10.3								

SD: standard deviation. ND: not determined.

Table S2. Analysis of TDF Pharmacokinetics and Pharmacodynamics in BLT and BALB/c Mice.

Mouse ID	TDF dose (mg/kg)	Time of harvest post dose (h)	Plasma TFV (ng/ml)	CVL TFV (ng/ml)	FRT TFV (ng/g)	FRT TFVdp (fmol/g)
BLT-300-1	300	24	374	1830	15680	1781059
BLT-300-2	300	24	377	16.0	5367	87129
BLT-300-3	300	24	427	8.70	3758	2925385
BLT-300-4	300	24	437	14.3	6912	2912142
BLT-300-5	300	24	246	31.6	4312	600447
BLT-300-6	300	24	41.5	48.2	923	35336
BLT-300-7	300	24	310	130	5097	3023969
BLT-300-8	300	24	705	9.70	2207	1959305
Median [IQR]			375.5 [135.5]	23.8 [55.5]	4704.5 [2383]	1870182 [2443335]
BALB/c-300-1	300	24	584	3940	10267	5219985
BALB/c-300-2	300	24	505	25.2	5979	2830475
BALB/c-300-3	300	24	328	3970	6710	1521538
BALB/c-300-4	300	24	372	298	6800	907308
BALB/c-300-5	300	24	338	2410	6031	3628989
BALB/c-300-6	300	24	445	1040	5050	2561521
BALB/c-300-7	300	24	371	5120	5220	3169703
BALB/c-300-8	300	24	539	717	4982	3582504
Median [IQR]			408.5 [150.75]	1725 [3335.25]	6005 [1555]	3000089 [1292600]

IQR: interquartile range

Table S3. Description of BLT mice Utilized to Evaluate TDF Pharmacokinetics and Pharmacodynamics.

		Peripheral blood humanization
Mouse ID	TDF dose (mg/kg)	%hCD4^{+} T cells of live cells
BLT-300-1	300	52.8
BLT-300-2	300	35.3
BLT-300-3	300	48.8
BLT-300-4	300	55.6
BLT-300-5	300	32.1
BLT-300-6	300	39.2
BLT-300-7	300	31.7
BLT-300-8	300	24.6
Mean ± SD		40 ± 11.2

SD: standard deviation

Table S4. Analysis of TDF Pharmacokinetics and Pharmacodynamics in BALB/c Mice.

Mouse ID	TDF dose (mg/kg)	Time of harvest post dose (h)	Plasma TFV (ng/ml)	CVL TFV (ng/ml)	FRT TFV (ng/g)	FRT TFVdp (fmol/g)
BALB/c-20-A	20	3	154	511	7861	288328
BALB/c-20-B	20	3	182	1530	8063	2559284
BALB/c-20-C	20	3	360	51.3	2929	1560291
BALB/c-20-D	20	3	177	363	2679	399595
BALB/c-20-E	20	3	239	284	2327	2404176
BALB/c-20-F	20	3	187	49.7	2173	1000424
BALB/c-20-G	20	3	217	50.2	5887	1385741
BALB/c-20-H	20	3	64.1	12900	855	222215
Median [IQR]			184.5 [51.25]	323.5 [714.725]	2804 [4092]	1193083 [1399484]
BALB/c-50-A	50	3	664	4.65	3581	896455
BALB/c-50-B	50	3	142	1.62	1979	733110
BALB/c-50-C	50	3	145	3.45	1956	1395973
BALB/c-50-D	50	3	106	4720	1777	544096
BALB/c-50-E	50	3	514	503	4126	2518406
BALB/c-50-F	50	3	461	29.6	125	94325
BALB/c-50-G	50	3	382	71800	8540	5219985
BALB/c-50-H	50	3	359	37700	3854	3039455
Median [IQR]			370.5 [330]	266.3 [12960.65]	2780 [2010.75]	1146214 [1962812]
BALB/c-140-A	140	3	1540	75200	15717	9450410
BALB/c-140-B	140	3	5460	16100	25420	7004474
BALB/c-140-C	140	3	2310	20400	7894	4189304
BALB/c-140-D	140	3	1440	67400	11875	9330808
BALB/c-140-E	140	3	1370	165000	12193	5479428
BALB/c-140-F	140	3	2040	9670	6063	4634426
BALB/c-140-G	140	3	1460	205	8287	9811564
BALB/c-140-H	140	3	1200	194000	21035	10636896
Median [IQR]			1500 [685]	43900 [83157.5]	12034 [8857.75]	8167641 [4272521]
BALB/c-300-A	300	3	2410	9360	23153	18695004
BALB/c-300-B	300	3	2430	210	15210	13872483
BALB/c-300-C	300	3	2530	2380	29869	7088469
BALB/c-300-D	300	3	2230	18700	19550	6568978
BALB/c-300-E	300	3	2200	390	21840	6117823
BALB/c-300-F	300	3	2410	871	21009	12842761
BALB/c-300-G	300	3	1130	9800	53130	947427
BALB/c-300-H	300	3	2440	43500	28855	2092282
Median [IQR]			2410 [210]	5870 [11274.25]	22496.5 [8464.25]	6828724 [7988754]

IQR: interquartile range
